# Assessing the Relationship between Foveal Cone Density, Outer Nuclear Layer Thickness and Foveal Morphology

**DOI:** 10.1016/j.xops.2025.100916

**Published:** 2025-08-18

**Authors:** Serena Zacharias, Joseph Kreis, Natalie Ungaretti, Emma Warr, Heather Heitkotter, Iniya Adhan, Ashleigh Walesa, Katherine Hemsworth, Jenna Grieshop, Joseph Carroll

**Affiliations:** 1 School of Medicine, Medical College of Wisconsin, Milwaukee, Wisconsin; 2 Department of Ophthalmology & Visual Sciences, Medical College of Wisconsin, Milwaukee, Wisconsin; 3 Department of Cell Biology, Neurobiology & Anatomy, Medical College of Wisconsin, Milwaukee, Wisconsin; 4 Division of Neurosurgery, Henry Ford Providence Southfield Hospital, Michigan State University, College of Human Medicine, Southfield, Wisconsin; 5 Joint Department of Biomedical Engineering, Marquette University and Medical College of Wisconsin, Milwaukee, Wisconsin

**Keywords:** Adaptive optics, Cones, Fovea, Foveal morphology, Outer nuclear layer

## Abstract

**Purpose:**

To assess the relationship between foveal cone topography, foveal outer nuclear layer (ONL) thickness, foveal morphology, and foveal avascular zone (FAZ) area in individuals with normal vision.

**Design:**

Retrospective cross-sectional study.

**Participants:**

A total of 68 participants with normal vision were included (49 female; 19 male).

**Methods:**

Directional OCT images were used to derive ONL thickness measurements. Images of the foveal cone mosaic were obtained using adaptive optics scanning light ophthalmoscopy, from which peak cone density (PCD) was measured. Foveal avascular zone area and foveal pit morphology were estimated using OCT angiography images and OCT macular thickness maps, respectively.

**Main Outcome Measures:**

Foveal cone density metrics, foveal ONL thickness, foveal pit diameter and volume, and FAZ area.

**Results:**

There was a weak positive correlation between maximum ONL thickness and PCD in individuals with normal vision (r = 0.23; *P* = 0.06), and PCD was significantly negatively correlated with both foveal pit diameter (r = –0.54; *P* < 0.0001) and foveal pit volume (r = –0.39; *P* = 0.0011).

**Conclusions:**

Findings suggest that foveal ONL thickness should be used with caution as a clinical biomarker of foveal cone density, at least when measured using current OCT technology. The relationship between foveal pit size and foveal cone density supports possible mechanistic links between the processes that establish these important features of foveal specialization.

**Financial Disclosure(s):**

Proprietary or commercial disclosure may be found in the Footnotes and Disclosures at the end of this article.

The fovea is a functionally important region of the retina with unique anatomical specializations relative to other retinal areas.^1^ These specializations include an avascular zone (termed the foveal avascular zone [FAZ]), an absence of inner retinal layers (defining the characteristic “pit”), specialized midget retinal ganglion cell circuitry, an increased packing density of cone photoreceptors, and an absence of rod photoreceptors in the central foveola. A number of retinal and systemic diseases are associated with altered foveal morphology, which is often accompanied by significant visual impairment. Characterization of the interrelationships between the various foveal specializations in these conditions offer an important opportunity to better understand their pathophysiology. In addition, such studies can provide insight into developmental mechanisms underlying key aspects of foveal specialization. However, in parallel to characterizing patients with retinal diseases, it is important to more thoroughly characterize foveal specializations in individuals with normal vision, especially given the advent of advanced high-resolution retinal imaging tools and the observation of remarkable variation in “normal” foveal anatomy.^2^^,^^3^

Of particular interest is outer nuclear layer (ONL) thickness measured from OCT, which is by far the most widely used metric to quantify photoreceptor structure.^4^, ^5^, ^6^, ^7^, ^8^ The extent to which ONL thickness measures agree with direct measures of cone density from adaptive optics scanning light ophthalmoscopy (AOSLO) and similar tools is variable and directly impacts the use of ONL thickness as a robust outcome measure in clinical trials and natural history studies. For example, Lee et al^9^ reported a positive correlation between foveal ONL thickness and peak cone density (PCD) in individuals with albinism, and Menghini et al^10^ also reported a positive correlation between ONL thickness and parafoveal cone density in individuals with normal vision and retinitis pigmentosa (although the 2 variables were also correlated with retinal eccentricity which makes predicting cone density from ONL thickness challenging). In contrast, Matlach et al did not observe a strong association between cone density and parafoveal ONL thickness, suggesting that there may be other determinants of ONL thickness beyond cone density.^11^ Additionally, Chui et al^12^ observed paradoxical age-related changes in these measures, with older individuals having thicker ONL values but decreased cone density values compared to younger individuals—concluding that ONL thickness may not be a sensitive marker of cone density.

Based on these disparate findings and the prior focus on parafoveal (not foveal) measures, there is a need to examine further the relationship between various anatomical specializations of the fovea. Here, we used AOSLO to image the foveal cone mosaic and directly test the hypothesis that foveal ONL thickness is correlated with foveal cone density. Additionally, we explored correlations between other foveal specializations including foveal pit morphology and FAZ area to assess how these parameters covary with cone density and ONL thickness at the fovea. Our results give important context for interpreting ONL thickness measures and provide data that may be useful in informing models of human foveal development.

## Methods

### Participants

This study followed the tenets of the Declaration of Helsinki and was approved by the Medical College of Wisconsin Institutional Review Board (PRO17439, PRO23898, PRO23999, PRO30741, and PRO31352). Participants were recruited from the local community using advertisements. After the nature and possible consequences of the study were explained, informed consent was obtained from all participants or from an appropriate adult guardian for minors. All participants provided an ocular health questionnaire and none reported vision limiting issues beyond corrective lenses. Some participants in this study have had portions of their data reported previously (Table S1, available at www.ophthalmologyscience.org).^13^ Adaptive optics scanning light ophthalmoscopy, OCT, and OCT angiography (OCTA) images from 68 participants (49 female; 19 male) with normal vision were included, ranging in age from 12 to 64 years (median = 26 years). Images acquired from the right eye were used unless only the left eye was imaged or right eye images were of poor quality. Prior to imaging, participants over the age of 18 were given 1 drop of phenylephrine hydrochloride (2.5%) followed by 1 drop of tropicamide (1%). Participants under the age of 18 were given 1 drop of cyclopentolate (1%). An IOL Master (Carl Zeiss Meditec) was used to measure axial length, which was used to correct the lateral scale of all retinal images.

### Adaptive Optics Scanning Light Ophthalmoscopy

Forty-five participants were imaged using a custom-built AOSLO system to acquire images of the foveal cone mosaic as described in a previous study.^13^ An additional 23 participants were prospectively recruited for this study. Using 790-nm or 680-nm light sources, images were collected using a 1° or 1.5° square raster. Participants were asked to fixate sequentially on 9 locations on the imaging raster, with a videos consisting of 150 to 200 frames being recorded at each location. To decrease variation in cone reflectivity, multiple videos were acquired at the same location at different time points or using different focus levels. For all videos, sinusoidal distortions were corrected using a previously described algorithm.^14^ Additionally, a previously described algorithm was used to automatically select a single reference frame against which the other frames in the video were registered.^15^ Then, the registered image sequences were averaged to produced images with a high signal to noise ratio. Using a previously described algorithm,^16^ a montage of the images was semiautomatically created using Adobe Photoshop CS6 (Adobe Systems, Inc.). After alignment of the images, a 300 × 300 μm region of interest centered on the fovea was extracted and overlapping images manually blended within Photoshop. Cones in this region were counted using a semiautomated algorithm (Mosaic Analytics, Translational Imaging Innovations) followed by review and confirmation by a single experienced observer (J.C.). These cone coordinates were used to created density maps using a sliding window method as previously described.^17^ The maximum density of cones in each foveal region of interest was considered the PCD. An 80% isodensity contour of the top 20th percentile densities was created, and the center of the contour was determined to be the cone density centroid (CDC) location.^18^ The code used to derive the density maps and extract the above metrics is available on GitHub (https://github.com/AOIPLab/Metricks/releases/tag/Zacharias_et_al_2025). To determine the areal foveal cone density, we aligned all density maps via the CDC point and found the common area as the maximum square distance in which all maps overlapped (0.227 mm). For each map, the horizontal row through the CDC point was extracted and averaged through the common area.

### Measuring ONL Thickness from Directional OCT Scans

On conventional OCT images, the Henle fiber layer (HFL) is often included in ONL thickness measures because the boundary between the 2 layers is difficult to distinguish. The HFL is comprised of photoreceptor axons and is oriented radially about the fovea because of the high density of cone photoreceptors and concomitant absence of inner retinal layers at the fovea. Importantly, measures of ONL thickness at one retinal location are “contaminated” by HFL structures emanating from other retinal locations, which obviates generating a simple offset to correct ONL thickness measurements at all retinal eccentricities. To determine the “true” ONL thickness, directional OCT (D-OCT) was used to identify the ONL/HFL boundary.^4^ Figure S1 (available at www.ophthalmologyscience.org) provides an overview of the HFL segmentation process, which is summarized below.

Using the Cirrus HD-OCT system (Carl Zeiss Meditec), 3 horizontal scans were obtained per eye, using nasal, central, and temporal pupil entry points. The scans were acquired using 6 mm or 9 mm (nominal length) high definition 5-line raster scans with 0–0.5 mm spacing, with the scan traversing the foveal center selected from each 5-line raster set. The actual lateral image dimension of each scan was determined by multiplying the nominal scan length (6 or 9 mm) by the ratio of the participant's measured axial length to the assumed axial length of the Cirrus device (24.46 mm). The central scan and the 2 off-axis B scans of each participant were aligned using a custom MATLAB (MathWorks) script.^19^ The triads of aligned D-OCT image sets were placed into a stack in ImageJ.^20^ Stacked images with minimal differences in the retinal pigment epithelium (RPE) contour, inner limiting membrane (ILM) contour, and choroidal vessels were considered sufficiently aligned, as assessed by 2 graders (S.Z. and N.U.). Grader 1 (S.Z) had prior experience in segmentation of D-OCT images, whereas grader 2 (N.U.) was newly trained in the segmentation process. The 3 aligned images were then merged using Z-project with max intensity, and the merged image was stacked with the triad of aligned scans. For 3 participants, choroidal vessels were not considered to be sufficiently aligned in one of the images, so only 2 images were merged. The resultant merged images were stacked with their respective input images so that they could be reviewed to ensure accurate delineation of the layer boundaries during the segmentation process. This was performed both graders using the point selection tool in ImageJ.^20^ Segmented boundaries include the RPE, the external limiting membrane, the interface between outer plexiform layer and HFL, the interface between HFL and ONL, and the ILM (as part of the standard segmentation process in our lab). Thirty segmentation points were applied per boundary for a total of 150 segmentation points. A custom MATLAB script (https://github.com/AOIPLab/Retinal_Thickness_Analysis/releases/tag/Zacharias_et_al_2025) was used to linearly interpolate the points on each boundary to obtain segmentation lines of the 5 boundaries. We then extracted ONL thickness (defined as the difference between the HFL/ONL boundary and the external limiting membrane) as a function of eccentricity from the foveal center. The maximum foveal ONL thickness was extracted, as well as the central foveal ONL thickness (calculated using the common area from the AOSLO data, 0.227 mm).

### Extracting Foveal Pit Metrics

Volumetric images (512 A-scans/B scan, 128 B-scans, nominally 6 × 6 mm) were acquired using the Cirrus HD-OCT system. The true lateral image dimension was again corrected for axial length by multiplying the nominal scan width by the ratio of the participant's measured axial length to the assumed axial length of the Cirrus device (24.46 mm). Topographical maps of retinal thickness (ILM-RPE thickness) were used to calculate foveal pit diameter and volume using a previously described algorithm.^21^, ^22^, ^23^ These volumes were also reviewed during initial screening and none of the participants had any visible pathological findings in these volumes.

### OCT Angiography Imaging

All participants were imaged with OCTA using the Optovue Avanti system (Optovue, Inc). One horizontal and one vertical scan (304 B-scans, 304 A-scans/B scan, nominal dimension = 3 × 3 mm) was acquired at the fovea and registered using the onboard software to create a single angiogram. Two to ten of these angiograms were acquired per eye as described in a previous study.^3^ An image “slab” from the ILM to 9 μm anterior to the outer plexiform layer was extracted from each angiogram. These images for each participant were then aligned using the ImageJ StackReg function^24^ and averaged using Z-project to create a single image per participant for analysis. Foveal avascular zone morphometry was extracted using a custom MATLAB script (https://github.com/AOIPLab/FIT). The algorithm works by identifying all parafoveal intercapillary areas (PICAs) within each image, with the largest central PICA being assigned as the FAZ. For some participants, their FAZ had a “fragmented” appearance, with capillaries traversing what would normally be a single well-demarcated avascular area. This was determined by calculating the ratio of each PICA in the central region to the largest PICA. Images with a single PICA ratio above 0.3 was determined to have a single FAZ, whereas images with multiple PICA ratios above 0.3 were deemed to have fragmented FAZs.^25^ For the participants determined to have a fragmented FAZ, the area of their largest central PICA was used as their FAZ area for analysis. See Figure S2 (available at www.ophthalmologyscience.org) for an example of a fragmented FAZ.

### Statistical Analysis

Statistical analyses were performed using Prism version 9 (GraphPad). Correlations within and between the various metrics (PCD, density at the CDC, areal average cone density, maximum ONL thickness, central ONL thickness, pit diameter, volume, and FAZ area) were assessed using Spearman correlation. Using R statistical software (Foundation for Statistical Computing), intraclass correlation coefficients were calculated for each grader's ONL thickness measurements. A paired *t* test was also computed on the ONL thickness measurements for the 2 graders.

## Results

The age of participants ranged from 12 to 64 years, and 72% (n = 49) were female. Axial length ranged from 22.18 to 27.49 mm (median = 24.02 mm), PCD ranged from 141 067 to 251 214 cones/mm^2^ (median = 191 211 cones/mm^2^, interquartile range (IQR) = 33 936 cones/mm^2^), whereas density at the CDC ranged from 135 948 to 244 368 cones/mm^2^ (median = 182 097 cones/mm^2^, IQR = 33 221 cones/mm^2^)—values for both metrics were consistent with previous reports.^13^^,^^26^, ^27^, ^28^ Areal foveal cone density ranged from 116 956 to 198 577 cones/mm^2^ (median = 153 808 cones/mm^2^, IQR = 27 174 cones/mm^2^). Foveal pit diameter ranged from 1.48 to 2.64 mm (median = 1.91 mm, IQR = 0.35 mm), whereas pit volume ranged from 0.02 to 0.19 mm^3^ (median = 0.09 mm^3^, IQR = 0.045 mm^3^)—again, these values are consistent with previous findings.^22^ A total of 6 of our 68 participants were found to have a fragmented FAZ, and across our cohort, the FAZ area ranged from 0.04 to 0.51 mm^2^ (median = 0.27 mm^2^, IQR = 0.16 mm^2^), which is also consistent with previous studies.^29^, ^30^, ^31^

Both graders showed moderate to good intergrader reliability for maximum foveal ONL thickness (intraclass correlation coefficient = 0.74, 95% confidence interval [CI] = 0.63-0.85) and central foveal ONL thickness measurements (intraclass correlation coefficient = 0.73, 95% CI = 0.62–0.84). However, the paired *t* tests showed small but significant differences between the 2 graders for both maximum foveal ONL thickness (t = 5.722, df = 67, *P* < 0.0001) and central foveal ONL thickness (t = 6.297, df = 67, *P* < 0.0001). As such, we used an average of Grader 1 and Grader 2's ONL thickness measurements for subsequent analyses. The average maximum foveal ONL thickness ranged from 74.13 to 131.60 μm (median = 97.63 μm), which is consistent with previous studies.^32^^,^^33^ The average central ONL thickness ranged from 74.30 to 128.37 μm (median = 98.30 μm).

**Figure 1:**
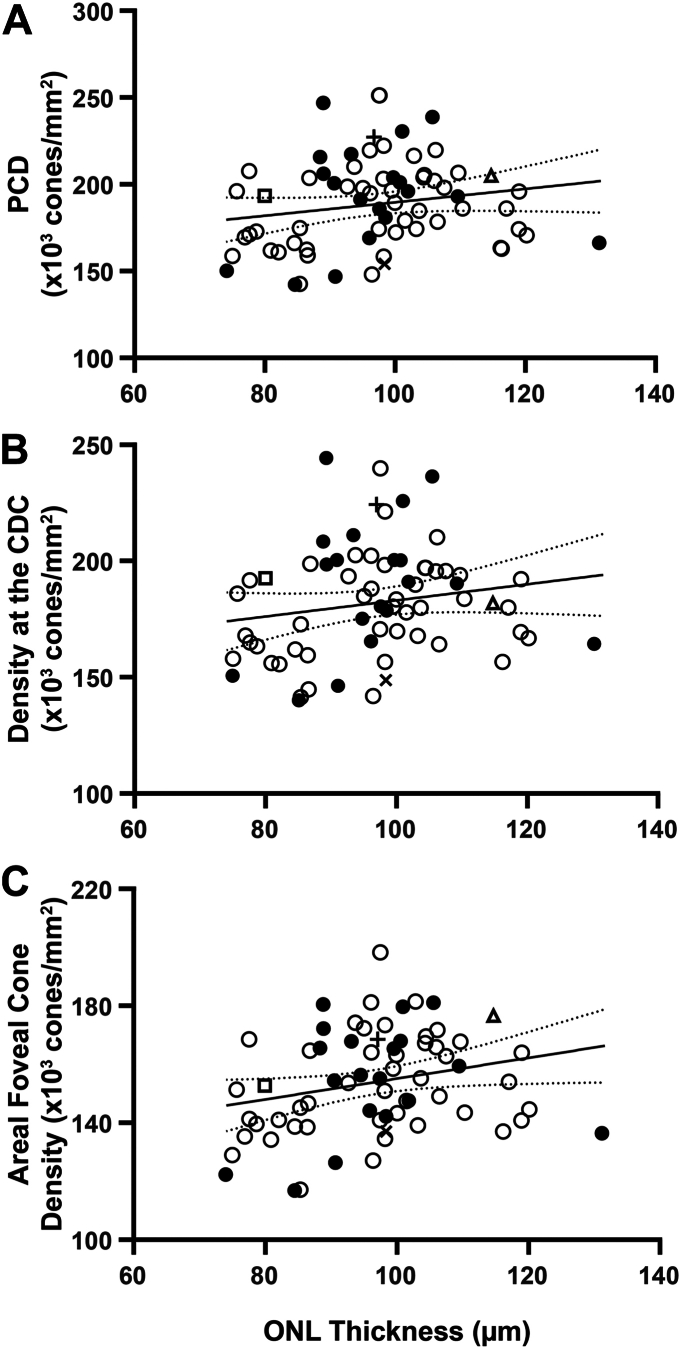
Assessing maximum ONL thickness against foveal cone density. **A,** There is a weak positive relationship between maximum ONL thickness at the fovea and PCD (r = 0.23; *P* = 0.06). **B,** There is also a weak positive relationship between maximum ONL thickness at the fovea and density at the CDC (r = 0.22; *P* = 0.08). **C,** There is a weak positive relationship between maximum ONL thickness at the fovea and areal foveal cone density (r = 0.26; *P* = 0.03). The solid line shows the Spearman correlation line, and the dashed lines represent the 95% confidence interval. Open symbols represent female participants, and closed symbols represent male participants. Square, triangle, cross, and x correspond to participants shown in Figure 2. CDC = cone density centroid; ONL = outer nuclear layer; PCD = peak cone density.

**Figure 2:**
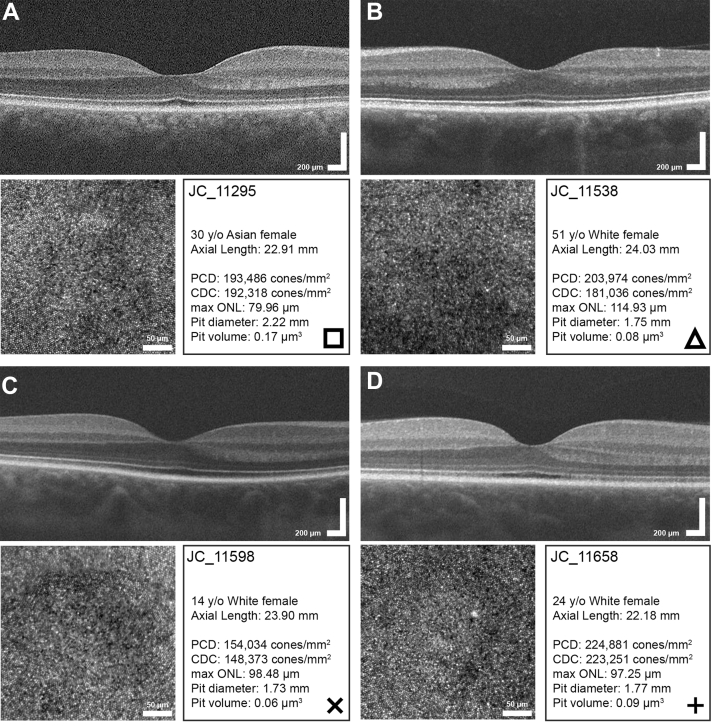
Comparing peak cone density and ONL thickness values. Panels **(A)** and **(B)** show D-OCT and AOSLO images of participants JC_11295, a 30-year-old Asian female and JC_11538, a 51-year-old White female, with similar peak cone densities and differing ONL thicknesses. Panels **(C)** and **(D)** show D-OCT and AOSLO images of participants JC_11598, a 14-year-old White female and JC_11658, 24-year-old White female, with comparable ONL thicknesses and varying peak cone densities. Symbols in the lower right of each panel represent the symbol used for that subject in Figures 1 and 3. AOSLO = adaptive optics scanning light ophthalmoscopy; D-OCT = directional OCT; ONL = outer nuclear layer; PCD = peak cone density.

**Figure 3:**
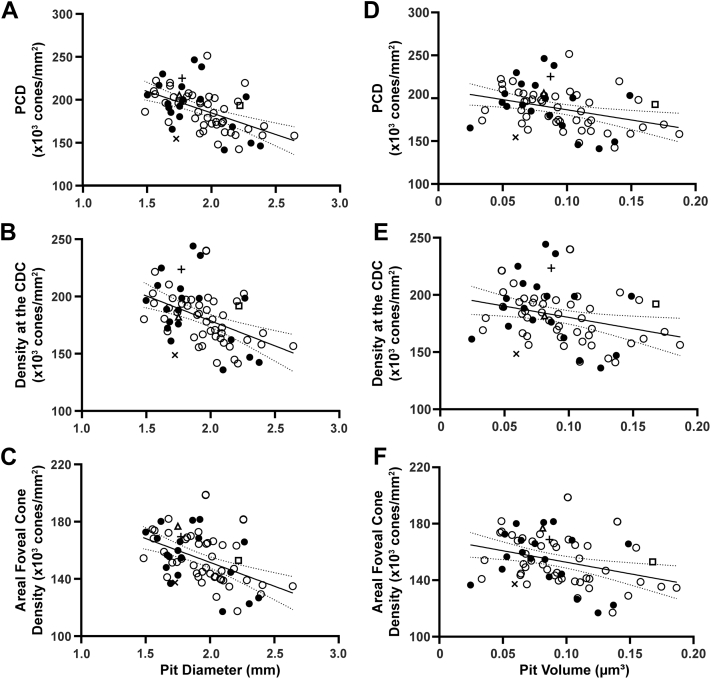
Assessing pit metrics against measures of foveal cone density. The plots show a negative correlation between **(A)** pit diameter and PCD (r = – 0.54; *P* < 0.0001), **(B)** pit diameter and density at the CDC (r = – 0.46; *P* < 0.0001) and **(C)** pit diameter and areal foveal cone density (r = –0.50; *P* < 0.0001). The negative correlation between **(D)** pit volume and PCD is also shown (r = –0.39; *P* = 0.0011) along with the negative correlation between **(E)** pit volume and density at the CDC (r = – 0.32; *P* = 0.0080) and the negative correlation between **(F)** pit volume and areal foveal cone density (r = –0.37; *P* = 0.0017). The solid line shows the Spearman correlation line, and the dashed lines represent the 95% confidence interval. Open symbols represent female participants, and closed symbols represent male participants. Square, triangle, cross, and x correspond to participants outlined in Figure 2. CDC = cone density centroid; PCD = peak cone density.

We observed weak positive correlations between average maximum ONL thickness and PCD (r = 0.23; *P* = 0.06), density at the CDC (r = 0.22; *P* = 0.08), and areal foveal cone density (r = 0.26; *P* = 0.03) (Fig 1). Similar relationships were seen when comparing the average central ONL thickness and PCD (r = 0.25; *P* = 0.04), density at the CDC (r = 0.24; *P* = 0.05), and areal foveal cone density (r = 0.28; *P* = 0.02) (data not shown). Worth noting, several participants had similar PCD values yet differing ONL thickness values, and others had comparable ONL thickness values with differing PCD values (Fig 2). The observed relationship between foveal size and both PCD and density at the CDC was of similar strength and direction (Fig 3). For example, pit diameter was negatively correlated with PCD (r = –0.54; *P* < 0.0001), density at the CDC (r = –0.46; *P* < 0.0001), and areal foveal cone density (r = –0.50; *P* < 0.0001). Similarly, pit volume was negatively correlated with PCD (r = –0.39; *P* = 0.0011), density at the CDC (r = –0.32; *P* = 0.008), and areal foveal cone density (r = –0.37; *P* = 0.0017). Foveal avascular zone area was strongly correlated with both pit diameter (r = 0.64; *P* < 0.0001) and pit volume (r = 0.67; *P* < 0.0001), consistent with results in previous studies.^2^^,^^21^

## Discussion

Developing reliable, validated biomarkers for assessing foveal cone structure is of great clinical importance. OCT-derived metrics are particularly attractive, given the widespread availability of OCT imaging devices. Here, we examined ONL thickness as a potential surrogate measure of foveal cone density in individuals with normal vision. We did not observe a significant positive correlation between measures of ONL thickness and measures of foveal cone density in our cohort, suggesting caution should be used when considering ONL thickness as a measure of cone numerosity (especially in cross-sectional studies). A key reason for this has to do with the composition of the ONL near the foveal—cone nuclei may be nonuniform in size, the presence of rod nuclei can contribute to ONL measures, and Müller glial cells might also affect ONL measures. Disambiguating between these biological explanations may require examination of animal models with varying rod or cone ratios, where histological comparisons would also be possible (and OCT and AOSLO measures are feasible in many animal models used in vision research). The extent to which ONL thickness can be used as a surrogate marker of cone density likely depends on multiple additional factors. For example, the use of D-OCT (or alternate modalities) to disambiguate ONL from HFL seems critical for the accuracy of ONL measures at any central retinal location. Moreover, if the location at which ONL is extracted does not align with the location of PCD, this would degrade the correlation between the 2 measures. Thus, it is worth noting that we derived of ONL thickness using a line scan—it is unknown whether the OCT B-scans were sliced through the foveal center precisely or whether it was coincident to the exact locus of PCD. Volumetric ONL data may allow for more accurate comparisons of foveal ONL thickness and foveal cone density topography (which is typically assessed over a larger area), although we did not see marked improvement in correlations when using the central ONL measurements that integrated thickness over the central 0.227 mm. Although such volumetric data can be extracted using similar D-OCT techniques as described here,^34^ this approach would be time consuming to apply on a larger scale. Polarization OCT techniques have also been shown to accurately demarcate the ONL and HFL layers over the macular region,^35^ and a recent volumetric D-OCT prototype was shown to provide efficient volumetric segregation of the HFL layer.^36^ The development of adaptive optics OCT technology also offers the possibility of collecting these measures simultaneously,^37^ which would ensure that measures of cone density and ONL thickness are extracted at the same retinal location. Future studies using these or other tools should permit expanded study of foveal ONL topography in larger cohorts and perhaps more precise coregistration of cone density and ONL thickness measures.

Because the inner and outer segments of photoreceptors are thought to degenerate before the nuclei in patients with inherited retinal degenerations,^38^ it seems likely that there could be a disconnect between cone density measures obtained using AOSLO and those derived from ONL thickness. As such, comparing the relationship between ONL thickness and cone density in patients with retinal degenerations could be valuable in understanding the pathophysiology of inherited retinal degeneration. Additionally, comparing a wider array of OCT-based measures of photoreceptor structure (such as outer segment length or ellipsoid zone intensity) to AOSLO measures of cone density could be informative in patients with inherited retinal degeneration. For example, it has been suggested that photoreceptor layer reflectivity on en face OCT correlates with cone photoreceptor density in patients with retinal disease.^39^ Similarly, reduced photoreceptor layer reflectivity is seen in patients with achromatopsia and cone dystrophy,^40^ although no direct measures of remnant cone structure were made in that study. Interestingly, in achromatopsia, there is not a correlation between foveal ONL thickness and remnant foveal cone inner segment density.^41^ Regardless, given the complexities of cone morphology and how it degrades in active disease, we suggest that longitudinal studies that incorporate direct measures of cone density on AOSLO and OCT measures of ONL thickness, photoreceptor layer reflectivity, and outer segment length will be informative not only in understanding the disease process but perhaps identifying limitations of these biomarkers. It is worth noting that direct imaging and quantification of cone nuclei may soon be achievable using OCT-based tools. Recent reports using a commercial “High-Res” OCT provide images showing putative cone nuclei.^42^ More promising are results from adaptive optics OCT, where cone nuclei can be more reliably resolved.^43^ It is not yet known how these measures correlate with conventional measures of OCT thickness, but such studies are well within reach.

The negative correlation observed between cone density and both pit diameter and pit volume are consistent with at least partially *coordinated* development of these anatomical foveal specializations, which are known to occur during overlapping time points in retinal development.^44^ However we previously observed that patients with albinism who had more well-developed foveal pits had higher foveal cone densities, whereas those with less well-developed pits had lower foveal cone densities,^22^ suggesting that a larger foveal pit further facilitates increased foveal cone packing. Although this may seem in conflict with the relationships we report here, it is worth noting that the pit-density relationship has been proposed to vary during development,^45^ so to the extent that albinism represents “arrested” development, these observations may not be contradictory. Additionally, central to these developmental models is the role of retinal stretch, which may differ between normal and albinotic populations.^46^ It is worth noting that there are data from multiple studies supporting at least partly independent developmental mechanisms underlying the various aspects of foveal specialization. First, the fact that patients with albinism and minimal foveal pit specialization have any degree of cone packing supports a separate active mechanism being involved in guiding foveal cone development. Additionally, we previously observed the presence of a normal FAZ in individuals with achromatopsia who lacked a fully formed foveal pit,^47^ decoupling 2 aspects of foveal anatomy that had previously been thought of as concomitant features of foveal specialization.^30^^,^^48^ Further evidence of separate developmental mechanisms comes from emerging data from other species showing that an area of high acuity (increased cone density and increased retinal ganglion cell density) can develop in the absence of a foveal pit.^49^^,^^50^ Prior OCT-based studies have provided insight into the postnatal aspects of foveal development,^51^, ^52^, ^53^ though they did not include measures of foveal cone topography. The emergence of handheld AO-based imaging tools^54^ should enable follow-up studies that monitor the evolution of all aspects of foveal specialization in humans.

There are additional limitations to our study beyond ones mentioned above. Cone density measurements for this study were obtained using cone coordinates validated by a single experienced grader. Although advances in AOSLO have improved the resolution of the photoreceptor mosaic, intergrader disagreement regarding the location of peak density have persisted due to the tight packing of cones at the fovea.^55^ A recent study demonstrated an error rate of 11.7% in PCD measurements across 5 graders with variable experience suggesting the importance of averaging multiple graders' estimates to determine a more accurate location for PCD.^56^ This is also relevant to studies aiming to correlate foveal cone topography with measures of visual function.^18^^,^^57^ Another major limitation to this study was the relatively homogenous population.A total of 56 of our 68 participants self-reported their race as white, and 49 out of 68 participants were female. Previous studies have reported sex and race related differences in retinal thickness and foveal pit morphology,^2^^,^^58^ so our results may not be applicable across all demographics.

Beyond applications for monitoring retinal disease or informing about foveal development, approaches like ours have implications for studying the aging retina. Histological data suggest that rods are more vulnerable than cones in normal aging.^59^ Because rod nuclei comprise the majority of the ONL thickness outside the central fovea, it is important to conduct similar studies comparing perifoveal ONL measures to underlying rod and cone density values from AO-based tools.^60^ Conducting such studies in patients with retinal degeneration could be useful to validate a recently proposed OCT approach to model photoreceptor degeneration.^61^ Ultimately, the ability to use noninvasive imaging to elucidate the anatomical integrity of human photoreceptors represents a powerful tool to support broader efforts to more effectively diagnose and treat patients with devastating retinal diseases.

## References

[1] Curcio C.A., Kar D., Owsley C. (2024). Age-related macular degeneration, a mathematically tractable disease. Invest Ophthalmol Vis Sci.

[2] Tick S., Rossant F., Ghorbel I. (2011). Foveal shape and structure in a normal population. Invest Ophthalmol Vis Sci.

[3] Ayala G.D., Linderman R.E., Valenzuela R.K. (2021). Assessing foveal structure in individuals with *TYR* R402Q and S192Y hypomorphic alleles. Ophthalmol Sci.

[4] Lujan B.J., Roorda A., Knighton R.W., Carroll J. (2011). Revealing Henle's fiber layer using spectral domain optical coherence tomography. Invest Ophthalmol Vis Sci.

[5] Cideciyan A.V., Jacobson S.G., Beltran W.A. (2013). Human retinal gene therapy for leber congenital amaurosis shows advancing retinal degeneration despite enduring visual improvement. Proc Natl Acad Sci USA.

[6] Huang W.C., Cideciyan A.V., Roman A.J. (2014). Inner and outer retinal changes in retinal degenerations associated with *ABCA4* mutations. Invest Ophthalmol Vis Sci.

[7] Takagi M., Maruko I., Yamaguchi A. (2019). Foveal abnormalities determined by optical coherence tomography angiography in children with history of retinopathy of prematurity. Eye.

[8] de Guimaraes T.A.C., Robson A.G., de Guimaraes I.M.C. (2024). CDH23-associated Usher syndrome: clinical features, retinal imaging, and natural history. Invest Ophthalmol Vis Sci.

[9] Lee D.J., Woertz E.N., Visotcky A. (2018). The henle fiber layer in albinism: Comparison to normal and relationship to outer nuclear layer thickness and foveal cone density. Invest Ophthalmol Vis Sci.

[10] Menghini M., Lujan B.J., Zayit-Soudry S. (2015). Correlation of outer nuclear layer thickness with cone density values in patients with retinitis pigmentosa and healthy subjects. Invest Ophthalmol Vis Sci.

[11] Matlach J., Mulholland P., Cilkova M. (2023). Ageing changes in retinal outer nuclear layer thickness and cone photoreceptor density using adaptive optics-free imaging. Eur J Ophthalmol.

[12] Chui T.Y., Song H., Clark C.A. (2012). Cone photoreceptor packing density and the outer nuclear layer thickness in healthy subjects. Invest Ophthalmol Vis Sci.

[13] Cava J.A., Allphin M.T., Mastey R.R. (2020). Assessing interocular symmetry of the foveal cone mosaic. Invest Ophthalmol Vis Sci.

[14] Dubra A., Harvey Z., Fischer B., Dawant B., Lorenz C. (2010).

[15] Salmon A.E., Cooper R.F., Langlo C.S. (2017). An automated reference frame selection (ARFS) algorithm for cone imaging with adaptive optics scanning light ophthalmoscopy. Transl Vis Sci Tech.

[16] Chen M., Cooper R.F., Han G.K. (2016). Multi-modal automatic montaging of adaptive optics retinal images. Biomed Opt Express.

[17] Warr E., Grieshop J., Cooper R.F., Carroll J. (2024). The effect of sampling window size on topographical maps of foveal cone density. Front Ophthalmol.

[18] Domdei N., Reiniger J.L., Holz F.G., Harmening W. (2021). The relationship between visual sensitivity and eccentricity, cone density and outer segment length in the human foveola. Invest Ophthalmol Vis Sci.

[19] Lujan B.J., Roorda A., Croskrey J.A. (2015). Directional optical coherence tomography provides accurate outer nuclear layer and henle fiber layer measurements. Retina.

[20] Schneider C.A., Rasband W.S., Eliceiri K.W. (2012). NIH image to ImageJ: 25 years of image analysis. Nat Methods.

[21] Dubis A.M., Hansen B.R., Cooper R.F. (2012). Relationship between the foveal avascular zone and foveal pit morphology. Invest Ophthalmol Vis Sci.

[22] Wilk M.A., McAllister J.T., Cooper R.F. (2014). Relationship between foveal cone specialization and pit morphology in albinism. Invest Ophthalmol Vis Sci.

[23] Wilk M.A., Dubis A.M., Cooper R.F. (2017). Assessing the spatial relationship between fixation and foveal specializations. Vis Res.

[24] Thévenaz P., Ruttimann U.E., Unser M. (1998). A pyramid approach to subpixel registration based on intensity. IEEE Trans Image Process.

[25] Linderman R.E., Cava J.A., Salmon A.E. (2020). Visual acuity and foveal structure in eyes with fragmented foveal avascular zones. Ophthalmol Retina.

[26] Zhang T., Godara P., Blanco E.R. (2015). Variability in human cone topography assessed by adaptive optics scanning laser ophthalmoscopy. Am J Ophthalmol.

[27] Wang Y., Bensaid N., Tiruveedhula P. (2019). Human foveal cone photoreceptor topography and its dependence on eye length. eLife.

[28] Domdei N., Ameln J., Gutnikov A. (2023). Cone density is correlated to outer segment length and retinal thickness in the human foveola. Invest Ophthalmol Vis Sci.

[29] Linderman R.E., Muthiah M.N., Omoba S.B. (2018). Variability of foveal avascular zone metrics derived from optical coherence tomography angiography images. Transl Vis Sci Tech.

[30] Ventrella D., Maya-Vetencourt J.F., Elmi A. (2022). The p-ERG spatial acuity in the biomedical pig under physiological conditions. Sci Rep.

[31] Otero-Marquez O., Haq A., Duran L.M. (2025). Preferential sites of retinal capillary occlusion in sickle cell disease. Invest Ophthalmol Vis Sci.

[32] Matsumoto H., Sato T., Kishi S. (2009). Outer nuclear layer thickness at the fovea determines visual outcomes in resolved central serous chorioretinopathy. Am J Ophthalmol.

[33] Mastey R.R., Gaffney M., Litts K.M. (2019). Assessing the interocular symmetry of foveal outer nuclear layer thickness in achromatopsia. Transl Vis Sci Tech.

[34] Kesim C., Bektas S.N., Kulali Z. (2022). Henle fiber layer mapping with directional optical coherence tomography. Retina.

[35] Motschi A.R., Schwarzhans F., Desissaire S. (2023). Characteristics of Henle's fiber layer in healthy and glaucoma eyes assessed by polarization-sensitive optical coherence tomography. Biomed Opt Express.

[36] Ni S., Khan S., Nguyen T.P. (2022). Volumetric directional optical coherence tomography. Biomed Opt Express.

[37] Jonnal R.S., Gorczynska I., Migacz J.V. (2017). The properties of outer retinal band three investigated with adaptive-optics optical coherence tomography. Invest Ophthalmol Vis Sci.

[38] Milam A.H., Li Z.Y., Fariss R.N. (1998). Histopathology of the human retina in retinitis pigmentosa. Prog Retin Eye Res.

[39] Saleh M., Flores M., Gauthier A.S. (2017). Quantitative analysis of photoreceptor layer reflectivity on en-face optical coherence tomography as an estimator of cone density. Graefes Arch Clin Exp Ophthalmol.

[40] Hood D.C., Zhang X., Ramachandran R. (2011). The inner segment/outer segment border seen on optical coherence tomography is less intense in patients with diminished cone function. Invest Ophthalmol Vis Sci.

[41] Langlo C.S., Patterson E.J., Higgins B.P. (2016). Residual foveal cone structure in *CNGB3*-associated achromatopsia. Invest Ophthalmol Vis Sci.

[42] Reche J., Stocker A.B., Henchoz V. (2023). High-resolution optical coherence tomography in healthy individuals provides resolution at the cellular and subcellular levels. Transl Vis Sci Tech.

[43] Kadomoto S., Muraoka Y., Uji A. (2021). Human foveal cone and Müller cells examined by adaptive optics optical coherence tomography. Transl Vis Sci Tech.

[44] Hendrickson A., Possin D., Vajzovic L., Toth C.A. (2012). Histologic development of the human fovea from midgestation to maturity. Am J Ophthalmol.

[45] Springer A.D., Hendrickson A.E. (2005). Development of the primate area of high acuity. 3: temporal relationships between pit formation, retinal elongation and cone packing. Vis Neurosci.

[46] Williams K.M., Georgiou M., Kalitzeos A. (2022). Axial length distributions in patients with genetically confirmed inherited retinal diseases. Invest Ophthalmol Vis Sci.

[47] Linderman R.E., Georgiou M., Woertz E.N. (2020). Preservation of the foveal avascular zone in achromatopsia despite the absence of a fully formed pit. Invest Ophthalmol Vis Sci.

[48] Chui T.Y.P., VanNasdale D.A., Elsner A.E., Burns S.A. (2014). The association between the foveal avascular zone and retinal thickness. Invest Ophthalmol Vis Sci.

[49] Beltran W.A., Cideciyan A.V., Guziewicz K.E. (2014). Canine retina has a primate fovea-like bouquet of cone photoreceptors which is affected by inherited macular degenerations. PLoS One.

[50] Choi J., Joisher H.N.V., Gill H.K. (2024). Characterization of the development of the high-acuity area of the chick retina. Dev Biol.

[51] Vajzovic L., Rothman A.L., Tran-Viet D. (2015). Delay in retinal photoreceptor development in very preterm compared to term infants. Invest Ophthalmol Vis Sci.

[52] Alabduljalil T., Westall C.A., Reginald A. (2019). Demonstration of anatomical development of the human macula within the first 5 years of life using handheld OCT. Int Ophthalmol.

[53] Bowl W., Stieger K., Bokun M. (2016). OCT-based macular structure-function correlation in dependence on birth weight and gestational age - the giessen long-term ROP study. Invest Ophthalmol Vis Sci.

[54] Hagan K., DuBose T., Cunefare D. (2020). Multimodal handheld adaptive optics scanning laser ophthalmoscope. Opt Lett.

[55] Putnam N.M., Hammer D.X., Zhang Y. (2010). Modeling the foveal cone mosaic imaged with adaptive optics scanning laser ophthalmoscopy. Opt Express.

[56] Wynne N., Cava J.A., Gaffney M. (2022). Intergrader agreement of photoreceptor topography at the foveal center on adaptive optics scanning light ophthalmoscopy. Biomed Opt Express.

[57] Rossi E.A., Roorda A. (2010). The relationship between visual resolution and cone spacing in the human fovea. Nat Neurosci.

[58] Wagner-Schuman M., Dubis A.M., Nordgren R.N. (2011). Race- and sex-related differences in retinal thickness and foveal pit morphology. Invest Ophthalmol Vis Sci.

[59] Curcio C.A. (2001). Photoreceptor topography in ageing and age-related maculopathy. Eye.

[60] Heitkotter H., Patterson E.J., Woertz E.N. (2022). Extracting spacing-derived estimates of rod density in healthy retinae. Biomed Opt Express.

[61] Whitmore S.S., DeLuca A.P., Androf J.L. (2023). Modeling rod and cone photoreceptor cell survival in vivo using optical coherence tomography. Sci Rep.

