# Supplementary material for: Assessing the Relationship between Foveal Cone Density, Outer Nuclear Layer Thickness and Foveal Morphology
Source: Ophthalmol Sci. 2025 Aug 18;6(1):100916. doi: 10.1016/j.xops.2025.100916 (PMC12548097; doi:10.1016/j.xops.2025.100916)
Supplement: Figure S1 [file mmc1.pdf]

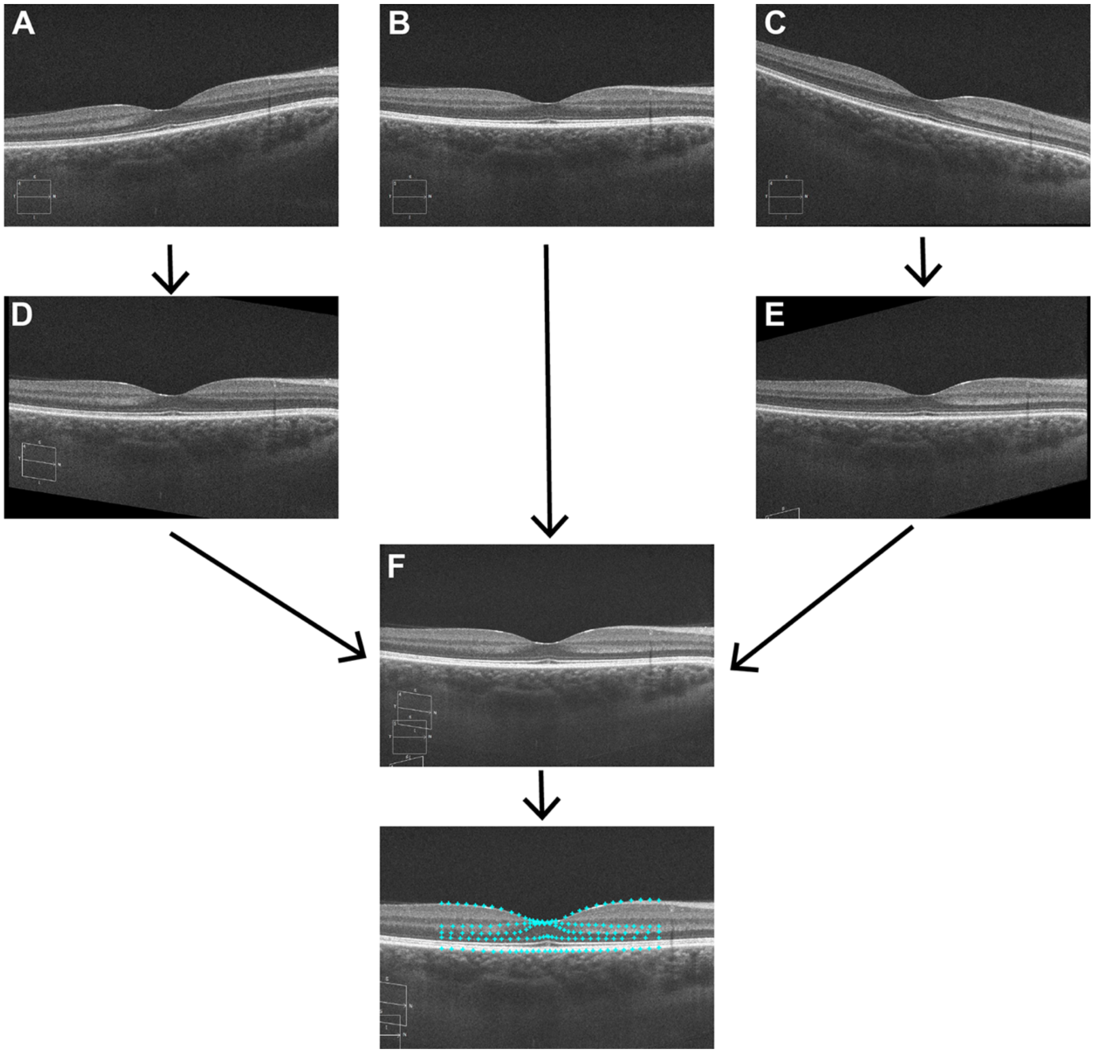

Supplemental Figure S1: Alignment and segmentation of D-OCT images. One central **(A)** and two off-axis scans **(B,C)** were acquired from each participant. All three scans were registered, and the off-axis scans were aligned. The central **(B)** and aligned off-axis scans **(D,E)** were merged to produce **(F)**. The merged image was stacked with the triad of aligned scans and were manually segmented on the merged image using ImageJ by two observers. Boundaries include the RPE, the external limiting membrane (ELM), the interface between outer plexiform layer (OPL) and HFL, the interface between HFL and ONL, and the ILM.
