# Supplementary figures and images for: Assessing the Relationship between Foveal Cone Density, Outer Nuclear Layer Thickness and Foveal Morphology
Source: Ophthalmol Sci. 2025 Aug 18;6(1):100916. doi: 10.1016/j.xops.2025.100916 (PMC12548097; doi:10.1016/j.xops.2025.100916)

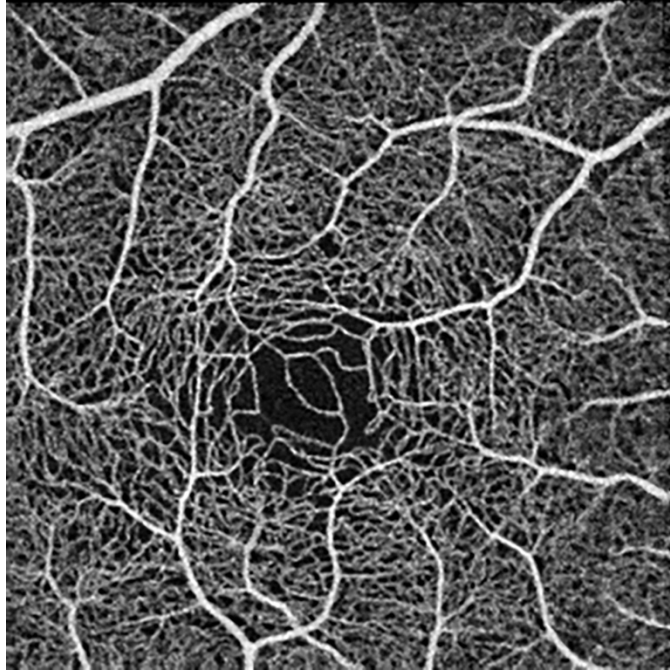

**Supplemental Figure S2:** Average OCTA image from participant JC\_11068, depicting a “fragmented” FAZ.

Supplement: Figure S2 [file mmc2.pdf]
